# X-Ray Scattering Reveals Two Mechanisms of Cellulose Microfibril Degradation by Filamentous Fungi

**DOI:** 10.1128/aem.00995-22

**Published:** 2022-08-23

**Authors:** Dimitrios Floudas, Luigi Gentile, Erika Andersson, Spyros G. Kanellopoulos, Anders Tunlid, Per Persson, Ulf Olsson

**Affiliations:** a Department of Biology, Microbial Ecology Group, Biology Department, Lund University, Lund, Sweden; b Dipartimento di Chimica and CSGI, Università degli Studi di Bari Aldo Moro, Bari, Italy; c Physical Chemistry, Lund University, Lund, Sweden; d National and Kapodistrian University of Athens, Faculty of Biology, Department of Genetics and Biotechnology, Athens, Greece; e Centre for Environmental and Climate Science, Lund University, Lund, Sweden; Royal Botanic Gardens

**Keywords:** Fenton chemistry, biodegradation, brown-rot fungus, cellulose, filamentous fungi, litter decomposer, white-rot fungus, X-ray scattering

## Abstract

Mushroom-forming fungi (*Agaricomycetes*) employ enzymatic and nonenzymatic cellulose degradation mechanisms, the latter presumably relying on Fenton-generated radicals. The effects of the two mechanisms on the cellulose microfibrils structure remain poorly understood. We examined cellulose degradation caused by litter decomposers and wood decomposers, including brown-rot and white-rot fungi and one fungus with uncertain wood decay type, by combining small- and wide-angle X-ray scattering. We also examined the effects of commercial enzymes and Fenton-generated radicals on cellulose using the same method. We detected two main degradation or modification mechanisms. The first characterized the mechanism used by most fungi and resembled enzymatic cellulose degradation, causing simultaneous microfibril thinning and decreased crystalline cellulose. The second mechanism was detected in one brown-rot fungus and one litter decomposer and was characterized by patchy amorphogenesis of crystalline cellulose without substantial thinning of the fibers. This pattern did not resemble the effect of Fenton-generated radicals, suggesting a more complex mechanism is involved in the destruction of cellulose crystallinity by fungi. Furthermore, our results showed a mismatch between decay classifications and cellulose degradation patterns and that even within litter decomposers two degradation mechanisms were found, suggesting higher functional diversity under current ecological classifications of fungi.

**IMPORTANCE** Cellulose degradation by fungi plays a fundamental role in terrestrial carbon cycling, but the mechanisms by which fungi cope with the crystallinity of cellulose are not fully understood. We used X-ray scattering to analyze how fungi, a commercial enzyme mix, and a Fenton reaction-generated radical alter the crystalline structure of cellulose. Our data revealed two mechanisms involved in crystalline cellulose degradation by fungi: one that results in the thinning of the cellulose fibers, resembling the enzymatic degradation of cellulose, and one that involves amorphogenesis of crystalline cellulose by yet-unknown pathways, resulting in a patchy-like degradation pattern. These results pave the way to a deeper understanding of cellulose degradation and the development of novel ways to utilize crystalline cellulose.

## INTRODUCTION

Cellulose is the most abundant carbohydrate (1) and a valuable resource for industrial applications (2). The primary structure of cellulose consists of simple linear chains of glucose residues connected with β-1,4-glycosidic bonds. However, the higher structure of cellulose is complex, comprising cellulose chains arranged together to form microfibrils, and the latter interact further to form cellulose fibers. Other important aspects of cellulose structure remain unclear; for example, the structural details of the cellulose microfibril surface, the differences in the degree of polymerization between amorphous and crystalline regions, and the presence and type of defects such as twists, kinks, or chain ends on the crystallites. Such aspects are important for the accessibility of cellulose to external reagents, such as microbial enzymes (3).

Fungi play an important role in cellulose degradation, and they employ two recognized degradation mechanisms. The first mechanism is enzymatic and involves the action of several enzymes with different functions (4, 5), including hydrolytic endoglucanases (EC 3.2.1.4), cellobiohydrolases (EC 3.2.1.91 and EC 3.2.1.176) (6, 7), and lytic polysaccharide monooxygenases (LPMOs; AA9 family), which cause oxidative cleavage of β-1,4 glycosidic bonds (5, 8) on crystalline cellulose. Enzymatic cellulose degradation is found across white-rot and soft-rot wood decomposers and also across litter decomposers (4). Nonenzymatic degradation of cellulose, which represents the second mechanism, is present in brown-rot fungi, which are members of the mushroom-forming fungi (*Agaricomycetes*). Brown-rot fungi secrete endoglucanases but no cellobiohydrolases (4, 9), and genomic analyses have shown that they have lost most or all enzymes that act on crystalline cellulose, including all cellobiohydrolases and most LMPOs (10). These findings represent a paradox, because in nature brown-rot fungi are efficient cellulose decomposers, leaving behind mostly partly oxidized lignin after wood decomposition is complete. Instead of the costly mechanism of enzymatic cellulose degradation, brown-rot fungi generate chelator-mediated Fenton-based radicals (11). The small size of the agents participating in the Fenton-based system allows them to diffuse into the wood matrix and cause cellulose oxidation, making it susceptible to depolymerization by endoglucanases (12).

Enzymatic degradation of cellulose by fungi has been mostly studied in wood decomposers and certain ascomycetes by detecting the degradation products, such as glucose, cellobiose, and oligosaccharides (13–17), providing valuable information about the release products and the efficiency of cellulose degradation under various conditions. Other studies have recognized that the structure of crystalline cellulose and the roughness of its surface influence the efficiency of degradation, and those researchers attempted to understand the interaction of enzymes with the microfibril surfaces (6, 18–20). In contrast, most aspects of the nonenzymatic mechanism remain poorly understood. As stated above, radicals produced by brown-rot fungi presumably oxidize cellulose, leading to the disruption of its crystallinity and making it susceptible to depolymerization by endoglucanases (11). However, whereas several studies have shown that this system can effectively depolymerize the amorphous regions of cellulose (21), only a few studies have provided evidence that it attacks crystalline cellulose (22–25). Furthermore, the cellulolytic mechanisms of litter decomposers remain largely unexplored, despite their important role in decomposition of litter and soil organic matter. For the reasons presented above, there is a need for techniques that will increase our understanding of the types of cellulose structure modification, from the nanoscale to roughly the scale of chemical bonds, caused by enzymatic and nonenzymatic fungal systems. Such techniques should facilitate comparative approaches and acquisition of data from multiple samples.

We used small- and wide-angle X-ray scattering (SAXS and WAXS), which have been used previously to study the structure of intact cellulose (26), to examine the changes in the structure of cellulose microfibrils caused by litter decomposers, white-rot and brown-rot fungi, and Schizophyllum commune, which exhibits an uncertain type of wood decay (27, 28). We also examined the effect of nitrogen availability in the degradation of cellulose by saprotrophic brown-rot wood decomposers in *Boletales*. The rationale behind the nitrogen experiment was that saprotrophic *Boletales* cause brown rot but still encode a moderate number of genes related to cellulose degradation (e.g., glycosyl hydrolase family 7 [GH7] proteins, LPMOs), suggesting that they could harbor both enzymatic and nonenzymatic cellulose degradation mechanisms (10, 29, 30). We hypothesized that under high-nitrogen conditions, brown-rot *Boletales* use the cellulolytic enzymes for cellulose degradation, while under low-nitrogen conditions they switch to the Fenton-based nonenzymatic system. As a comparison for the nitrogen experiment, we used Gloeophyllum trabeum, which does not produce cellobiohydrolases (10). We compared fungal cellulose degradation to that caused by commercial enzymes and an *in vitro* Fenton reaction. We found evidence for the presence of two cellulose microfibril degradation types, one of which agrees with enzymatic degradation (thinning) and another that is connected to cellulose amorphogenesis (patchy-like) but did not match the effect of Fenton-based radicals. Moreover, the two mechanisms did not match the current ecological or wood decay classifications that separate mushroom-forming fungi into white-rot and brown-rot wood decomposers and litter decomposers, suggesting that functional diversity among the three groups is more complex than we currently think.

## RESULTS

### Scattering features of crystalline cellulose.

**FIG 1:**
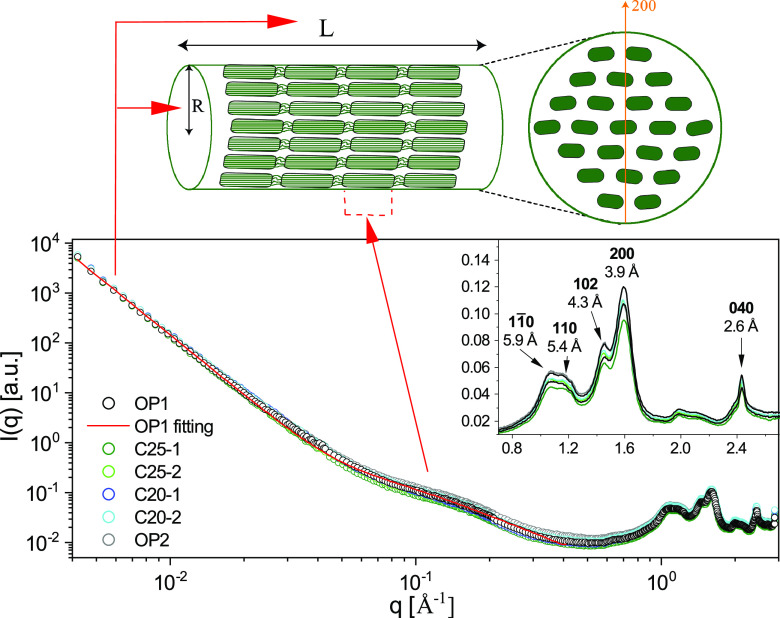
Full scattering profiles for the original filter paper (two replicates each of OP1 and OP2) and the control filter paper sample (two replicates each of C25-1, C25-2, C20-1, and C20-2), incubated for 40 days at 25°C and 20°C. The association of the scattering regions with the relevant structure of a cellulose fiber is shown. The red line was obtained by fitting the OP1 SAXS profile with equation 1 in the range 0.004 to 0.45 Å^−1^. The intensity was normalized for the transmitted beam. The absolute intensity was obtained from the OP thickness, 0.430 mm. The inset highlights WAXS results and shows the main reflections of cellulose I. The numbers in the inset represent Miller indices (57).

Changes in cellulose structure were investigated by using SAXS and WAXS covering almost 3 orders of magnitude in the scattering vector, *q*, from 0.004 to 2.9 Å^−1^. The various cellulose treatments examined are listed in Table 1. Fig. 1 shows the SAXS and WAXS patterns and scattering intensity [*I*(*q*)] of the original (nonautoclaved, nonincubated) filter paper (OP), together with control filter papers (autoclaved), incubated for 40 days in the liquid medium without fungi. The six different patterns showing the scattering intensity over the *q* range [*I*(*q*)] were identical, and the scattered intensity and the *I*(*q*) profile were reproducible. This suggests that the sterilization process and incubation of the autoclaved paper in the absence of fungi did not alter the fiber structure of cellulose. Therefore, we used the pattern from the OP as a reference for investigating the impact of the treatments.

**TABLE 1:** Fungal strains, nutritional strategy (NS), and incubation temperature during the experiments^*a*^

Sample	Acronym	NS	Incubation temp (^o^C)
Original paper (nonautoclaved)	OP		
Autoclaved paper	AUP		
Control 20C	C20		20
Control 25C	C25		25
*Agrocybe pediades* CBS 102.39 (G)	AGP	LD	25
*Coprinellus angulatus* CBS 175.51 (G)	COP	LD	20
*Gymnopus confluens* CBS 406.79 (G)	GYC	LD	20
*Leucoagaricus leucothites* CBS 146.42 (G)	LEL	LD	25
*Mycetinis scorodonius* CBS 850.87	MYS	LD	25
*Tetrapyrgos nigripes* CBS 291.85 (G)	TEN	LD	25
*Tricholomella constricta* CBS 661.87 (G)	TRC	LD	20
*Psilocybe* cf *subviscida* CBS 101986 (G)	PSS	LD	20
*Gloeophyllum* sp. OMC-1627	GLO	BR	25
*Boletales* sp. FD-575-SV (LN)	BOL	BR	20
*Coniophora puteana* RWD-64-598 ss2 (G) (LN)	CP	BR	20
*Fomitopsis pinicola* FD-585	FP585	BR	25
*Gloeophyllum trabeum* ATCC 11539 (G) (LN)	GTG	BR	25
*Hydnomerulius pinastri* MD-312 (G) (LN)	HP	BR	20
*Phanerochaete* sp. FD-574	PHL	WR	25
*Bjerkandera adusta* HHB-12826 (G)	BA	WR	25
*Ceriporiopsis subvermispora* FP-105752 strain B (G)	CS	WR	25
*Schizophyllum commune* FD-588 A	SCA	UWD	25

a LD, litter decomposer; WR, white rot; BR, brown rot; UWD, uncertain wood decay type. (G) indicates that the genome sequence of the strain is published. (LN) indicates strains that were used in both high-nitrogen and low-nitrogen experiments.

The overall scattering pattern *I*(*q*) had the following features: (i) At lower *q* values (0.004 to 0.04 Å^−1^), the intensity decayed as a power law, *I*(*q*) = *Aq*^−4^, with *A* being a constant. (ii) At an intermediate *q* of ≈0.1 Å^−1^, there was a small bump in the scattering curve. (iii) In the high-*q* WAXS regimen (*q* > 0.8 Å^−1^), the diffraction pattern demonstrated the presence of crystalline cellulose phases I_α_ and I_β_ (31, 32), which are polymorphs of cellulose I. The peaks labeled in the inset of Fig. 1 indicate the Miller indices of the crystallographic plane of I_β_, whereas for I_α_ we would expect 100, 010, 110, 123, and 114 (33). The two polymorphs were indistinguishable in the WAXS data, but they were distinguishable by solid-state nuclear magnetic resonance (34).

We interpreted the low *q* power law as the overall scattering of the fibers, *I*_fib_(*q*) = *Aq*^−4^. As the fiber diameter was much greater than a *q*_min_^−1^ of ≈250 Å (*q_min_*, ≈0.004 Å^−1^), the *q*^−4^ decay of the scattered intensity can be described in terms of Porod’s law (35), where the prefactor, *A*, is proportional to the total fiber interfacial area, *S*, exposed to the X-ray beam (Fig. 1). We attributed the bump in the scattering curve at *q *of ≈0.1 Å^−1^ to the internal structure of the fibers (Fig. 1). Previous work concluded that this bump corresponds to a peak associated with the packing of cellulose microfibrils, but its exact origin is debated (26, 36, 37). We describe this feature with a simple Gaussian function, *I*(*q*)_int_ = *B*[exp(−*kq*^2^) + *bg*], where *k* is a constant. Assuming that this structural feature is homogeneous throughout the fibers, *bg* is a *q*-independent (background) contribution associated with the nonperiodic part of the local molecular packing. The WAXS part of the overall pattern, for *q* of >0.8 Å^−1^, with a series of Bragg peaks, indicates mainly the presence of crystalline domains in the fiber. Cellulose degradation results in a decrease in the intensity, while the diffraction pattern remains constant. We can thus describe the intensity as *I*_cr_(*q*) = *CS*_cr_(*q*), where the structure factor *S*_cr_(*q*) describes the diffraction pattern and *C* is a measure of the diffracted intensity, which is proportional to the amount of crystalline cellulose. Assuming that the crystalline domains are homogeneously distributed in the fibers, then *C*, just like *B*, is proportional to the total fiber volume in the X-ray beam.

The SAXS region can be modeled as follows:

(1) $$I\left(q\right)\;=I_{\text{fib}}\left(q\right)\;+\;I_{\text{int}}\left(q\right)\;=Aq^{-\text{4}}\;+\;Be^{-kq^{2}}+\;\mathrm{bg}$$

A best fit to the SAXS curve of OP in the *q* range of 0.004 to 0.4 Å, using equation 1, is shown as a solid line (Fig. 1). As can be seen, the SAXS data were well described by the model. This kind of fit allows for determining the proportionality factors *A* and *B*. As a measure of the factor *C*, in the WAXS region, we used the peak intensity of the most intense Bragg peak (Miller index of 200) in the diffraction pattern, at *q *= 1.63 ± 0.04 Å^−1^. The full scattering profiles were normalized considering the sample transmission, *T*. Thus, *T* is equal to:

(2) $$T=\frac{I_{t}}{I_{i}}=e^{-\left(\frac{\mu }{\rho }\right)d\delta }$$

Where *I_i_* and *I_t_* are the intensities of incident beam and transmitted beam, respectively, δ is the thickness, and *d* is the sample density, while the ratio μ/ρ is the mass attenuation. For small *d*δ:

(3) $$1-T=1-e^{-\left(\frac{\mu }{\rho }\right)d\delta }\approx \left(\frac{\mu }{\rho }\right)d\delta$$

**FIG 2:**
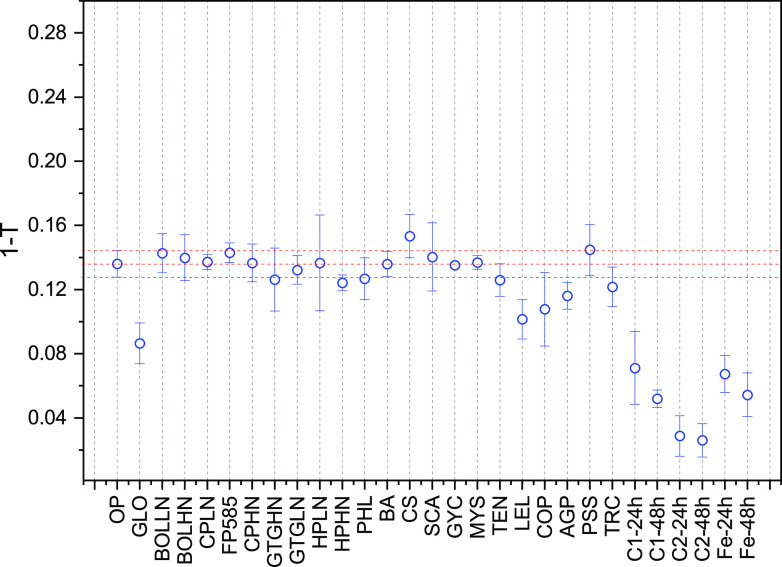
Transmission (*T*) values for all samples. *T* values were obtained by averaging the transmission value of each pattern collected at the three sample-to-detector distances and divided by the average value for the original paper (OP).

Thus, the change in *T* can also be used as measure of material loss, as nontransmitted X-rays (1 − *T*) are either adsorbed or scattered. For OP, *T *is 0.86, and in Fig. 2 we plotted 1 − *T* as obtained from the different experiments. The transmission here was relatively high, so that (1 − *T*) was approximately proportional to the total volume of scatterers. *Gloeophyllum* sp. OMC-1627, *Leucoagaricus leucothites* (LEL), *Coprinellus angulatus* (COP), *Agrocybe pediades* (AGP), and enzymatic and Fenton treatments revealed lower values of (1 − *T*), consistent with a higher material loss.

### Effect of commercial cellulolytic enzymes and Fenton reaction radicals on cellulose structure.

**FIG 3:**
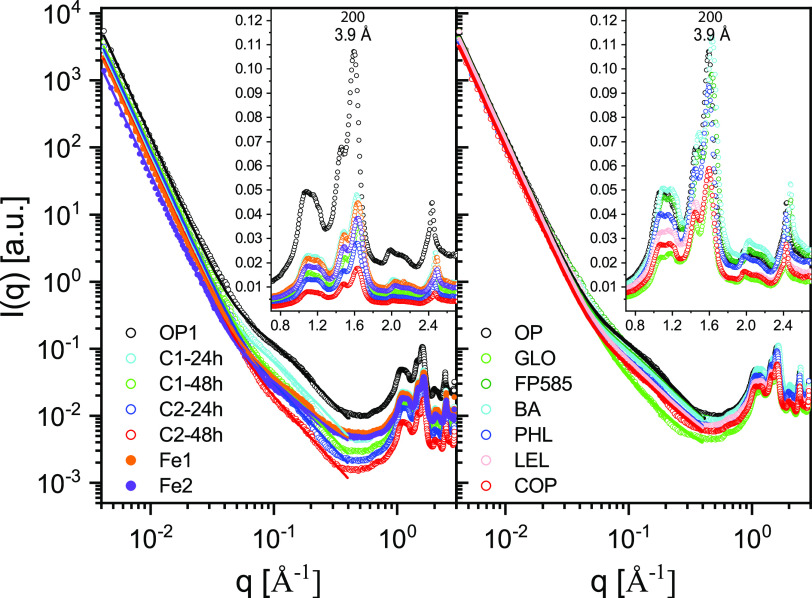
Full scattering profiles of the original filter paper and following exposure to enzymes, the Fenton reaction, and fungal species. (Left) Full scattering profiles of the original filter paper (OP), the filter paper after 24- and 48-h exposure to commercial enzyme concentrations C1 and C2 (C1-24h, C1-48h, C2-24h, and C2-48h), and after exposure to the Fenton reaction radicals (Fe1, Fe2). (Right) Full scattering profiles of OP and after fungal degradation by selected species (see Table 1 for species abbreviations). The data are reported along with the model (equation 1). The insets for both panels highlight the WAXS region.

**FIG 4:**
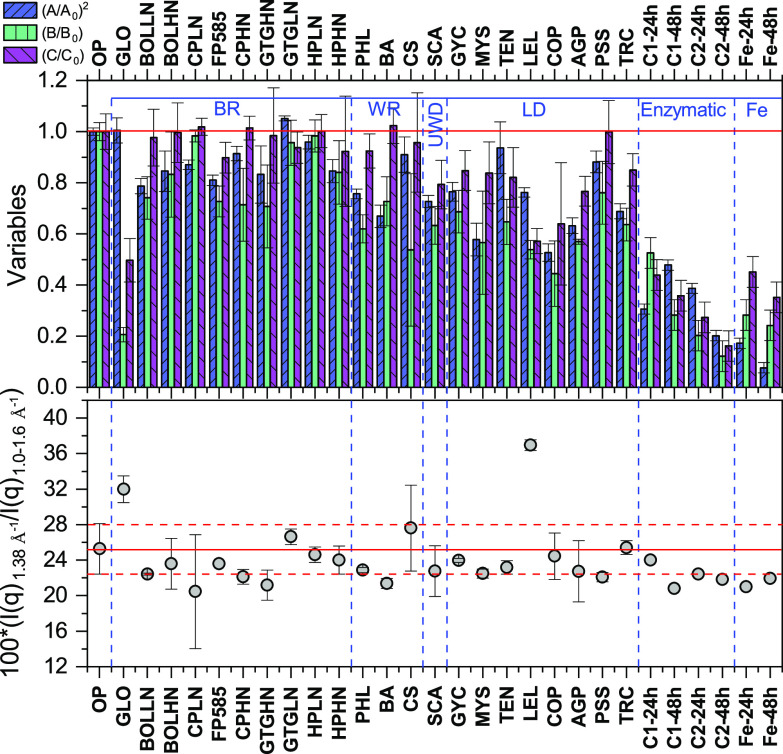
SAXS and WAXS fitting parameters and estimates of amorphous cellulose. (Top) The *A* and *B* fitting parameters of equation 1 relative to the data in Fig. 1 and 3 and the square root of the *C* parameter obtained at the *q* position of 1.63 ± 0.04 Å^−1^. *A* and *B* were normalized for the values obtained from the OP (*A_0_* and *C_0_*, respectively). (Bottom) Calculated ratios between the scattering intensity at the expected position of the amorphous peak at 1.38 Å^−1^ and the sum of all the intensities of the peaks between 1 and 1.6 Å^−1^, including the intensity at 1.38 Å^−1^ (40). The intensity was obtained by deconvolution (see the “Additional description of analyses” in the supplemental material). The red line represents the average values obtained from the original samples, the autoclaved samples, and the control samples at 20°C and 25°C. The dotted lines mark the areas of relative deviation. LD, litter decomposer; WR, white rot; BR, brown rot; UWD, uncertain wood decay strategy; Fe, Fenton reaction. Species names corresponding to the acronyms are shown in Table 1. LN and HN after the acronym indicate species that were incubated in medium with low or high nitrogen content, respectively.

**FIG 5:**
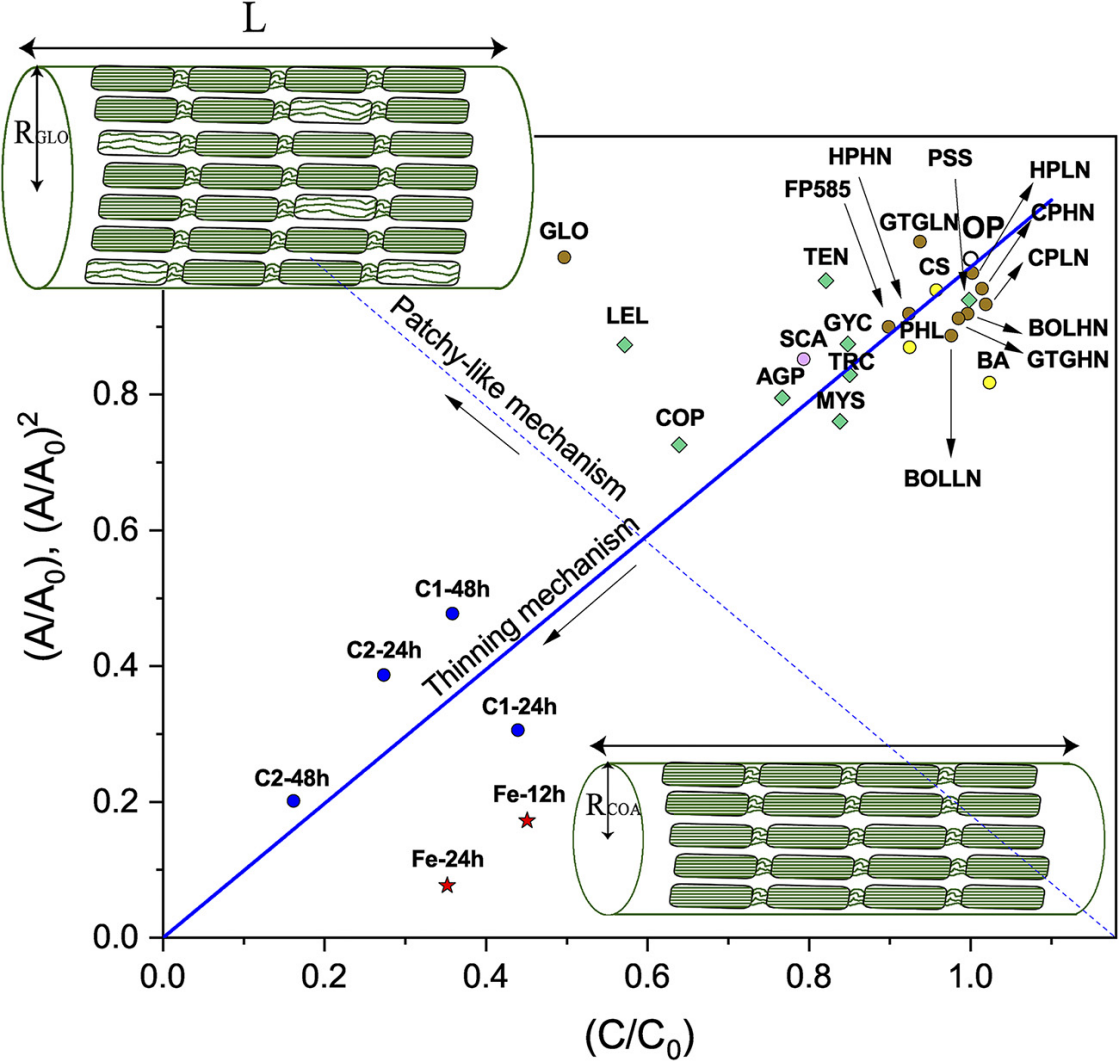
Relationship between overall fiber volume, proportional to (*A*/*A*_0_)^2^, and the total volume of crystalline domains, proportional to *C*/*C*_0_. The relationship between (*A*/*A*_0_)^2^ and *C*/*C*_0_ provides a schematic summary of the two types of cellulose degradation mechanisms detected in this study. *Gloeophyllum* sp. and *Leucoagaricus leucothites* cause melting of the crystallites and generation of amorphous cellulose without concomitant thinning of the fibrils. We refer to this as the patchy-like mechanism. In contrast, *Coprinellus angulatus* uses an enzymatic degradation mechanism during which the fibrils are degraded from their surface inwards with simultaneous, proportional loss of crystalline cellulose, which we refer to as a thinning mechanism. We assume for both these mechanisms that the number of fibers remain constant on the experimental time scale. *A*/*A*_0_ versus *C*/*C*_0_ was plotted in the case of enzymatic (C1-24h, C1-48h, C2-24h, C2-48h) and Fenton (Fe-12h, Fe-24h) degradation, due to a decrease in scattering (fiber) volume at a constant fiber radius, *R*. Colors stand for different ecologies or treatments: yellow, white-rot fungi; brown, brown-rot fungi; green, litter decomposers; pink, uncertain wood decay type; blue, enzymatic treatments; red, Fenton treatments; open circles, control sample.

Using the original filter paper as a reference, with *A* = *A*_0_, *B* = *B*_0_, and *C* = *C*_0_, we characterized the cellulose degradation and modification patterns caused by fungi (Fig. 3, right), the commercial enzymatic mix, and the Fenton reaction radicals (Fig. 3, left), in terms of the relative values *A/A_0_*, *B/B_0_*, and *C/C_0_* (Fig. 4a and 5). The values of *A*, *B*, and *C* extracted from the corresponding SAXS and WAXS profiles (Fig. 2) were normalized for the reference value of the OP. Fig. 4a shows (*A/A_0_*)^2^, *B/B_0_*, and *C/C_0_* values or all the investigated samples. As mentioned above, if the fibers were homogeneous in crystallinity, then C would be expected to be proportional to the fiber volume. In contrast, *A* is proportional to the interfacial area, and in the case of cylindrical fibers, to the fiber radius *R*. Thus, for cylindrical fibers we expect *A*^2^ to be proportional to the fiber volume. This means that if the effect of cellulose degradation were a homogeneous thinning of the fibers, then we would expect to have (*A/A_0_*)^2^ = *C/C_0_*. And, if the average fiber radius decreased from the initial value, *R_0_*, to *R*, then *R*/*R_0_* = *A/A_0_* = (*C/C_0_*)^1/2^. The crystallinity of cellulose filter papers of this kind is typically high: a value of 76% was reported for filter paper similar to the one used in this study (38). The intensity at *q *= 1.38 Å^−1^ is considered to have a significant contribution from amorphous cellulose (38–40) (see Fig. S1 in the supplemental material). To assess whether the ratio (*α*) between amorphous and crystalline cellulose varied across treatments, we calculated the ratio between the intensity at *q *of 1.38 Å^−1^ and the sum of peak intensities in the *q* range of 1 to 1.6 Å^−1^ (Fig. 4b; see also Fig. S1 in the supplemental material). Although we could not quantitatively analyze the absolute value of *α*, we hypothesized that variations in *α* reflect variations in the crystallinity, i.e., *α* increases if the crystallinity decreases.

The enzymatic treatment of cellulose at two different enzyme concentrations resulted in a significant decrease of *I*(*q*) over the entire *q* range (Fig. 3, left) and a subsequent decrease of all three calculated ratios (Fig. 4a). Higher enzyme concentrations (C1, C2) and longer incubation times (24 h, 48 h) resulted in larger decreases in the SAXS and WAXS intensities. Moreover, we observed (*A/A_0_*)^2^ ≈ *C/C_0_*, consistent with a homogeneous thinning of the fibers (Fig. 5), with the radius *R*/*R*_0_ decreasing to ≈0.45 after 48 h of incubation with the higher enzyme concentration. We also estimated a slight decrease of the ratio of amorphous to crystalline cellulose (Fig. 4b), which suggests that amorphous cellulose is more accessible than crystalline cellulose and thus easier to decompose (5). Finally, transmission values were higher than for all other samples, indicating a smaller amount of cellulose remained (see the “Additional description of analyses” in the supplemental material). Similar to the enzymatic treatment, the Fenton treatment resulted in a decrease of the *I*(*q*) over the entire *q* range (Fig. 3, left), with a subsequent strong decrease of all three ratios (Fig. 4a). The comparison between the two incubation times showed that most of the degradation due to the Fenton reaction happened in the first 24 h. We also observed a slight decrease of *α* (Fig. 4b), which suggested that again the amorphous cellulose was more prone to degradation (41, 42). In contrast to the enzymatic treatment, the Fenton treatment resulted in a disproportional decrease between (*A/A_0_*)^2^ and (*C/C_0_*) (Fig. 5). This result implies that while the fibers became thinner (*A/A_0_*), the amount of crystalline cellulose did not decrease proportionally to the radius, which further suggests fundamental differences between the mode of action of enzymes and Fenton reaction radicals during cellulose degradation. It should also be mentioned that in the case of both enzymatic (C1-24h, C1-48h, C2-24h, and C2-48h) and Fenton (Fe-12h and Fe-24h) degradation, the loss of material was so large that the number of fibers was most likely decreased. In the case that the fibers were degraded one by one, this would result in a gradual decrease of the filter paper thickness, which would then correspond to a decrease in scattering (fiber) volume at constant fiber radius, *R*. Therefore, we expect *A*/*A*_0_~*C*/*C*_0_ (Fig. 5). For these experiments *A*/*A*_0_~*C*/*C*_0_ fits better, and we have for these cases plotted *A*/*A*_0_ versus *C*/*C*_0_.

### Efficiency of fungal cellulose degradation.

Three species of white-rot fungi, six species of brown-rot fungi, eight species of litter decomposers, and *S. commune* (uncertain wood decay type), all representing four orders (*Agaricales*, *Polyporales*, *Boletales*, and *Gloeophyllales*) were examined for their ability to degrade or modify cellulose fibers without nitrogen limitation (Table 1). In addition, four of the brown-rot species were cultivated on cellulose under low-nitrogen conditions (Table 1). The degree of cellulose degradation differed among the examined fungi, as shown by the *I*(*q*) profiles (Fig. 3, right) and the changes in the calculated ratios (Fig. 4a and 5). The observed differences were not related to the two different incubation temperatures (Table 1). Overall, all wood decayers, independently of their wood decay type (except for *Gloeophyllum* sp. OMC-1627), showed similar efficiency in degrading cellulose. This was obvious from all calculated values of *A/A_0_*, *B/B_0_*, and *C/C_0_* (Fig. 4a and 5), which decreased to a similar degree. The different nitrogen concentrations (high N [HN] and low N [LN]) did not have a large impact on the thinning of cellulose fibers (based on *A/A_0_* and *C/C_0_*); however, we observed a more pronounced decrease of *B/B_0_* under high-nitrogen conditions (Fig. 4a), particularly for *Gloeophyllum trabeum*, *Coniophora puteana*, and *Hydnomerulius pinastri*. In contrast to wood decayers, litter decomposers were in general more efficient in decomposing cellulose, as shown by the *A/A_0_* and *C/C_0_* values. Within litter decomposers, we observed a larger variation in cellulose degradation efficiency, with the strains of *Leucoagaricus leucothites* and *Coprinellus angulatus* being the most efficient ones. These results were confirmed by the transmission measurements (see the “Additional description of analyses” in the supplemental material).

### Mechanistic insights into cellulose degradation by fungi.

Independently of the substrate type (litter versus wood) or wood decay strategy (white versus brown rot), most fungi had a similar effect on cellulose structure. Similarly, the two different nitrogen concentrations did not alter the patterns of cellulose degradation by brown-rot *Boletales*, which implies that nitrogen might not have a regulatory effect under these experimental conditions. For most samples, the relationship of (*A/A_0_*)^2^ ≈ *C/C_0_* was maintained, i.e., there was a decrease of the overall volume *V* of the fibers (thinning) (Fig. 5) and a proportional decrease in the volume of crystalline cellulose. This effect resembled the enzymatic degradation of cellulose, and it was in agreement with the rich repertoire of cellulose-degrading enzymes in litter decomposers and white-rot fungi (43–45) and with studies showing that cellulolytic enzymes can cause peeling of the fibers (6, 18, 19). Furthermore, for most fungi there was an equal preference between crystalline and amorphous cellulose or a slight preference for amorphous cellulose, as shown by the slight change of α (Fig. 4b). The strongest thinning effect was seen for *Coprinellus angulatus*, which codes for a rich repertoire of genes related to crystalline cellulose degradation and could be adapted to substrates enriched in crystalline cellulose (43). The thinning of the cellulose fibrils was also seen for most brown-rot fungi and was similar to that measured for white-rot fungi, contrary to our expectation that the strong genomic differences between white-rot fungi and litter decomposers versus brown-rot fungi would be depicted by the effect that the latter group of fungi had on cellulose structures (43). Our findings suggest that the effect of brown-rot fungi on cellulose results in a similar thinning of the fibers, while it is different from the effect that the *in vitro* Fenton reaction has on cellulose.

The strain of the brown-rot fungus *Gloeophyllum* sp. and the strain of the litter decomposer *Leucoagaricus leucothites* were the only two strains that did not agree with the findings described above. Both strains showed an unusual pattern of cellulose degradation that did not match the enzymatic or Fenton treatments. Cellulose degradation by both species manifested itself in a strong decrease in the diffraction peak intensities (*C/C_0_ =* 0.5) and the intermediate peak intensity (*B/B_0_* = 0.3) (Fig. 4a). In contrast, the decrease of the intensity in the low-*q* power-law regimen was not so prominent (Fig. 4a). As discussed above, we would expect an *A/A_0_* equal to *R/R_0_*, as *A* is strictly proportional to the area of interface. However, an increase in the surface roughness during cellulose degradation by these species could explain the discrepancy between the minor decrease in *A* and the significant decrease in fiber volume, as seen by the decrease of *C*. Another possible explanation could be that there is a decrease in crystallinity without significant cellulose depolymerization. This would be the case if *Gloeophyllum* sp. and *L. leucothites* employed mechanisms that disrupted the crystallinity of cellulose, transforming it into its amorphous form, which would render it susceptible to further depolymerization (Fig. 5). This apparent “melting” of crystallites is supported by the observed increase in the α ratio (Fig. 4b). Although most species appeared to decompose amorphous and crystalline cellulose at the same rate, both *Gloeophyllum* sp. and *L. leucothites* behaved differently, since α was observed to increase by ≈50% for *L. leucothites* and 30% for *Gloeophyllum* sp. This indicated a patchy modification mechanism, since the overall structure of the fibers was maintained but amorphous material increased, suggesting that only some crystallites were exposed to amorphogenesis.

## DISCUSSION

### Implications for fungal mechanisms of cellulose degradation through the eyes of X-ray scattering.

Earlier studies showed that brown-rot and white-rot fungi have different effects on cellulose, particularly on its degree of polymerization, but only a few studies have examined their effects on the crystallinity of cellulose (22–25). Even less is known about the effect that litter decomposers have on cellulose. Accumulating genomic data also support the dichotomy between white-rot and brown-rot fungi and the overall similarity of litter decomposers to white-rot fungi at the genomic level regarding their cellulolytic gene content (43). The use of X-ray scattering in this study revealed a disagreement between genomic data and laboratory experiments. We detected two main cellulose degradation mechanisms. The first one matched enzymatic cellulose degradation, leading to a gradual thinning of the cellulose fibers and a simultaneous removal of crystalline and amorphous cellulose (Fig. 4b and 5). This mechanism was detected for most examined species, including white-rot and brown-rot fungi and litter decomposers. The second mechanism, which was caused only by one brown-rot fungus (*Gloeophyllum* sp.) and one litter decomposer (*L. leucothites*), led to a decrease of crystallinity without proportional thinning of the fibers (Fig. 5), resulting in accumulation of cellulose with amorphous characteristics (Fig. 4b).

The apparent melting of crystalline cellulose and the molecular mechanisms underlying its generation by *Gloeophyllum* sp. and *L. leucothites* are not well understood. Although brown-rot fungi utilize crystalline cellulose in wood, they lack most of the enzymes that are known to be involved in crystalline cellulose degradation (4, 10, 29, 46). It has been suggested that brown-rot fungi employ a chelator-mediated Fenton system that generates radicals involved in degradation of cellulose and other carbohydrates (11, 25). Such a system could explain the results we report here for *Gloeophyllum* sp., and they agree with recently published data based on Raman spectroscopy (43). Furthermore, such a mechanism could explain the preferential loss of genes involved in the degradation of crystalline cellulose and the retention of endoglucanases seen across brown-rot lineages (10). However, our results also suggest that amorphogenesis of cellulose is not caused by all brown-rot fungi. This could be related to differences between the degradation mechanisms across brown-rot fungi (47) or the environmental cues that regulate such mechanisms, but also additional factors such as the amount of time since the strain has been isolated in culture and whether the strain is dikaryotic or monokaryotic (48–51). Moreover, cellulose amorphogenesis caused by *Gloeophyllum* sp. and *L. leucothites* does not seem to be merely the result of the Fenton reaction for two reasons. First, the SAXS and WAXS patterns from the Fenton treatment were different from those recovered from *Gloeophyllum* sp. and *L. leucothites*. A possible explanation is that *in vitro* the Fenton reaction is nonspecific, whereas the one generated by fungi causes a controlled but poorly understood (and possibly spatially targeted) production of hydroxyl radicals in close proximity to cellulose. Second, the cellulolytic gene composition of *L. leucothites* showed similarity to white-rot fungi (43), and therefore we should not expect that a Fenton-based system is active in *L. leucothites*. Alternatively, this could imply the presence of dual enzymatic and nonenzymatic systems in *L. leucothites*, such as those present in saprotrophic *Boletales* (e.g., *Hydnomerulius pinastri*, *Coniophora puteana*), which cause brown rot, but they also code for enzymes involved in crystalline cellulose degradation (4, 29, 52). Dual systems that involve enzymes and Fenton radicals involved in lignin degradation have been previously reported in white-rot fungi (53). Moreover, we cannot exclude that *L. leucothites* employs other less-known mechanisms, such as the action of expansins (54). Another important factor that has to be taken into consideration in light of these results is the incubation time. Here, we sampled only one time point (40 days). While we consider this to be an adequate time lapse for the extensive modification of cellulose by the fungi, it represents only a glimpse of the fungal effect on cellulose. Therefore, a deeper understanding of the molecular mechanisms involved in cellulose amorphogenesis will require complementary experimental approaches that will combine time-resolved transcriptomic experiments with the X-ray scattering methods employed in this study. Moreover, the extent to which the amorphogenesis mechanism is regulated by environmental factors needs to be examined more thoroughly. Here, we examined the effect of nitrogen for a small set of brown-rot species, but future studies should include additional species and more diverse growth conditions, as well as more complex types of colonized samples, such as wood. It should also be mentioned that while the X-ray scattering analysis is suitable for moderately decomposed cellulose samples, it becomes challenging in heavily degraded samples, as discussed above for the enzymatic and Fenton reaction experiments, because of the significant thinning of the samples, which in turn interferes with the analysis of the data and the interpretation of the results.

A better understanding of fungal cellulose degradation is also necessary because of its role in terrestrial carbon cycling and its connection to industrial applications (2). While WAXS has been used previously to examine the structure of intact cellulose microfibrils (26), SAXS and WAXS have not been used together to examine the effect of Fenton reaction, commercial enzymes, and saprotrophic fungi on the structure of cellulose. Our results show that this combination yields reproducible scattering data offering information on two different levels. From a mechanistic point of view, we showed that it is feasible to examine the degradation of the individual states of cellulose (e.g., amorphous versus crystalline) and therefore to better understand the structures targeted by the different degradation mechanisms. Such information cannot be obtained by measuring the cellulolytic products (e.g., glucose) only. The data obtained here can be further used to explore the potential applications of fungal mechanisms that cause amorphogenesis of cellulose in industrial applications related to the paper pulp and biofuel industries. From an ecological point of view, we have provided qualitative estimates of the efficiency of degradation, and we show that degradation differs across and even between ecological groups, even when the examined species share the enzymes for cellulose degradation (43). This points to differences in the regulation of those genes and/or the efficiency of the encoded enzymes (55), and also the possible role of little-known mechanisms in cellulose degradation by fungi. The current study demonstrates the power of X-ray scattering to shed light into cellulose degradation mechanisms, the factors that regulate them, and their distribution across fungi.

## MATERIALS AND METHODS

### Experimental design.

The study aimed to understand changes in the structure of cellulose microfibrils during degradation by strains of mushroom-forming fungi with diverse ecologies, by commercially produced cellulolytic enzymes, and by Fenton-generated hydroxyl radicals. For this reason, we incubated selected fungal strains using high-crystallinity cellulose as the sole carbon source for 40 days, providing other nutrients in the form of a liquid medium. In separate experiments, we incubated high-crystallinity cellulose in solutions containing commercial enzymes and separately in solutions that promoted the generation of Fenton reaction hydroxyl radicals. For all experiments, a sample of cellulose was excised and used to record SAXS and WAXS data.

### Strains and culture conditions.

The experiments included eight litter decomposers and four white-rot and six brown-rot wood decomposers (Table 1). Ηigh-quality filter paper (Whatman, grade 2589a; diameter of 140 mm, thickness of 0.43 mm) was used as cellulose source. The paper was placed in 150-mm petri dishes with 18 mL of modified Highley medium (43), which contained all necessary nutrients and ammonium nitrate (2 g/liter) as nitrogen source but no additional carbon source. For four of the brown-rot species, we additionally examined their ability to degrade cellulose under low-nitrogen conditions (NH_4_NO_3_ at 0.01 g/liter). In addition to the nitrogen source, the Highley medium contained (per liter): 2 g KH_2_PO_4_, 0.5 g MgSO_4_ · 7H_2_O, 0.1 g CaCl_2_ · 2H_2_O, 0.57 mg HBO_3_, 0.036 mg MnCl_2_ · 4H_2_O, 0.31 mg ZnSO_4_ · 7H_2_O, 0.039 mg CuSO_4_ · 5H_2_O, 0.018 mg (NH_4_)_6_Mo_7_O_24_ · 4H_2_O, 0.015 mg FeSO_4_ · 7H_2_O, and 10 mL of a vitamin stock solution. The vitamin stock solution contained (per liter): 1 g inositol, 0.01 g thiamine-HCl, 0.0025 g biotin, 0.01 g pyrodoxine, 0.01 g riboflavine, 0.01 g nicotinamide, 0.01 g *p*-aminobenzoic acid, 0.01 g Ca-panthotenate. A Nylon 66 membrane (polyamide; pore diameter of 1 μm) was placed on top of the paper, and an inoculum (7 mm) from actively growing cultures of the strains was placed at the center of each dish. The cultures were incubated at 20 or 25°C (Table 1). After 40 days, the membrane and the remaining liquid were removed, and the filter papers were dried overnight at 50°C.

### Commercial enzymes and Fenton reaction treatments.

The enzymatic treatment of cellulose was done using the commercial preparation Cellic CTec2 (Novozyme). Nine or 18 μL of the enzymatic mix was added in a final volume of 10 mL sodium acetate buffer (0.1 M, pH 4.8) to form concentrations C1 and C2, respectively. Filter paper (100 mg) was incubated for 24 and 48 h at 50°C under rotation (280 rpm). At the end of the incubation, the liquid was removed and the paper was washed twice with MilliQ water and dried at room temperature for 24 h. Earlier experiments had shown that these condition are suitable for the advanced degradation of filter paper cellulose (43). Cellic CTEC2 was selected as the most suitable commercial enzymatic preparation, since it contains GH6, GH7, LPMO, and β-glucosidase activities (16, 56). White-rot fungi and litter decomposers in *Agaricomycetes* secrete the same types of enzymes for cellulose degradation (4, 10).

The Fenton reaction was performed in 5 mL sodium acetate buffer (0.2 M; pH 4) at room temperature. The final concentrations of freshly prepared Fe^2+^ and hydrogen peroxide were 0.005 M and 0.8 M, respectively. The surplus of hydrogen peroxide was used to ensure a continuous Fenton reaction for 48 h. The pH was measured every hour for the first 6 h and at 8, 10, 12, 14, 16, 19, and 24 h. It remained close to 3.65 throughout the experiment. After 24 h, the liquid was removed and the paper was washed twice with MilliQ water and dried at room temperature for 24h.

### SAXS and WAXS.

Paper samples, of approximately 0.5 cm by 0.5 cm, before and after fungal degradation were placed in a “sandwich” sample holder without windows. All measurements were performed under vacuum. The sample holder was scanned by means of transmission experiments to locate accurately the sample positions. The sample was taken about 1 cm away from where the fungal inoculum had been placed to ensure prolonged exposure of the cellulose to the fungus. SAXS and WAXS measurements were performed using a SAXSLab Ganesha 300XL instrument (SAXSLAB ApS, Skovlunde, Denmark), which includes a pinhole-collimated system equipped with a Genix 3D X-ray source (Xenocs SA, Sassenage, France). The scattering intensity *I*(*q*) was recorded with the detector placed at three sample-to-detector distances, yielding scattering vectors (*q*) from 0.004 to 2.9 Å^−1^. Samples were located in a sandwich cell holder (Hilgenberg GmbH, Malsfeld, Germany). The temperature was controlled by an external recirculating water bath fixed to 25°C (accuracy of ca. 0.2°C). The two-dimensional (2D) scattering pattern was recorded using a 2D 300 k Pilatus detector (Dectris Ltd., Baden, Switzerland) and radially averaged using SAXSGui software to obtain *I*(*q*). The SAXS and WAXS data were normalized by the intensity of the transmitted beam through the sample, whereas thickness was fixed to 0.430 mm, i.e., the thickness of the original paper (OP). The overall thickness of the decomposed filter paper changed very little with respect to the OP, making any measurement of the thickness unsuitable. The deconvolution of the WAXS patterns is described in Fig. S1 in the supplemental material. The SAXS-WAXS method was chosen over other methods because it allowed us not only to estimate the crystallinity, but also to evaluate fiber thinning.

### Data availability.

All data are available in the main text or the supplemental material.

The raw scattering data are available upon request.
