# Supplementary material for: X-Ray Scattering Reveals Two Mechanisms of Cellulose Microfibril Degradation by Filamentous Fungi
Source: Appl Environ Microbiol. 2022 Aug 23;88(17):e00995-22. doi: 10.1128/aem.00995-22 (PMC9469724; doi:10.1128/aem.00995-22)
Supplement: Supplemental file 1 — Additional description of analyses. Download aem.00995-22-s0001.pdf, PDF file, 0.8 MB [file aem.00995-22-s0001.pdf]

Supplementary Material

All X-ray scattering data generated during the experiments are found in the data file SAXS_Cellulose.dat.

WAXS analysis and deconvolution

In the wide-angle WAXS regime, 0.5 $Å^{-1}$< q < 3 $Å^{-1}$ we observe the diffraction pattern from the crystalline domains of cellulose I, which can be assigned to the β polymorph based on Figure 1. As a measure of the diffraction intensity we recorded the 200 peak intensity at q=1.63±0.04 $Å^{-1}$, and referred to this intensity as C. Furthermore, it has been shown that purely amorphous cellulose appears in the WAXS regime as a broad peak centered at 1.38 $Å^{-1}$ (1). In Figure 3 (right panel) we calculated the ratio between the area of the peak at 1.38 $Å^{-1}$, and the total area of all WAXS peaks, coming from the crystalline cellulose, between 1 and 1.6 $Å^{-1}$ (2, 3).

$$100 \cdot \frac{A(q)_{1.38}}{[A(q)_{1.07}+A(q)_{1.18}+A(q)_{1.38}+A(q)_{1.48}+A(q)_{1.63}]} \qquad \text{(eq. S1)}$$

The deconvolution of the WAXS patterns was performed considering the Gaussian function as the shape of the resolved peaks at 1.07±0.04 $Å^{-1}$, 1.18±0.04 $Å^{-1}$, 1.48±0.04 $Å^{-1}$ and 1.63±0.04 $Å^{-1}$ while the amorphous peak was fixed at 1.38 $Å^{-1}$.

$$I(q) = B(q) + \sum_{i=1}^{5} \frac{A_i e^{\frac{-4\ln(2)\left(q-q_{c,i}\right)^2}{w_i^2}}}{w_i\sqrt{\frac{\pi}{4ln(2)}}} \qquad \text{(eq. S2)}$$

where $B(q)$ is the background intensity that was for each deconvolution as the minimum intensity after peak 200 (1.63±0.04 $Å^{-1}$), $q_{c,i}$, is the center of each peak i, A_i is the area, and w_i is full width at half maximum that was fixed to 0.4 for the amorphous peak at 1.38 $Å^{-1}$.

The areas of the peaks were obtained by deconvolution of the WAXS region between 0.84-0.95 Å^{-1} and 1.81-1.95 Å^{-1} and one pattern is presented as an example in Figure S1.

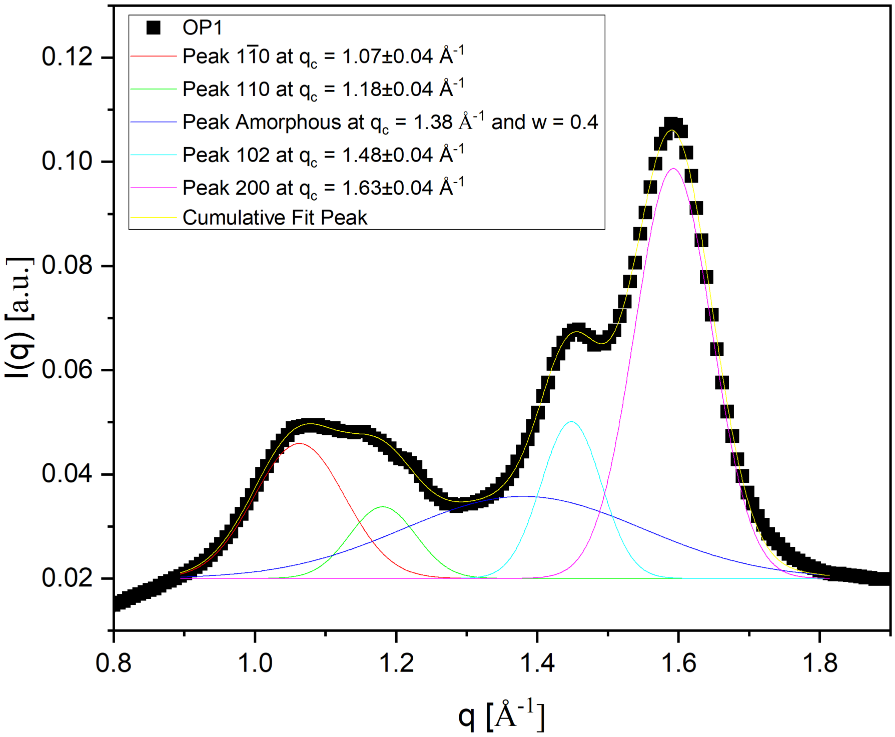

Figure S1. Deconvolution of WAXS patterns for the original paper (OP).

The deconvolution analysis is considered a way to estimate the ratio of amorphous to crystalline cellulose, eq. S2 in a sample. However, there are several assumptions and mathematical analyses that can lead to a wide uncertainty on the final value. In our case, only the relative differences were discussed. The full width at half maximum of the peak 200 was (0.12 ± 0.02) Å^{-1}.

1. Zugenmaier P. X-ray analysis of partial crystalline fibre structure [Internet]. Vol 1, Handbook of Textile Fibre Structure. Woodhead Publishing Limited; 2009. 46–120 bl. Available at: http://dx.doi.org/10.1533/9781845696504.1.46

2. Hall M, Bansal P, Lee JH, Realff MJ, Bommarius AS. Cellulose crystallinity - A key predictor of the enzymatic hydrolysis rate. FEBS J. 2010;277(6):1571–82.

3. Fernandes AN, Thomas LH, Altaner CM, Callow P, Forsyth VT, Apperley DC, et al. Nanostructure of cellulose microfibrils in spruce wood. Proc Natl Acad Sci U S A. 2011;108(47):E1195–E1203.
